# Our Co-Laborers

**Published:** 1877-02-20

**Authors:** 


Our Co-Laborers. — A number of able and intelligent medical men have engaged to write for our journal the present year. Their names were unintentionally omitted from the proper place, and will appear in our next. Our subscribers will please remember that, though they are not all on our published list of co-laborers, yet we regard them as such, and shall be pleased to have them send us up useful and practical matter for publication at any time.

